# A case-cohort study of human herpesvirus 8 seropositivity and incident prostate cancer in Tobago

**DOI:** 10.1186/1750-9378-6-25

**Published:** 2011-12-07

**Authors:** Alicia C McDonald, Frank J Jenkins, Clareann H Bunker, John W Wilson, Alan L Patrick, Joel L Weissfeld

**Affiliations:** 1 Department of Epidemiology, Columbia University, 722 West 168th Street, 720A, New York, New York, 10032, USA; 2 Department of Pathology, University of Pittsburgh, HCCLB Room G.17, 5117 Centre Avenue, Pittsburgh, Pennsylvania, 15232, USA; 3 Department of Epidemiology, University of Pittsburgh, A542 Crabtree Hall, 130 DeSoto Street, Pittsburgh, Pennsylvania, 15261, USA; 4 University of Pittsburgh Cancer Institute, UPMC Cancer Pavilion, Suite 4-C, 5150 Centre Avenue, Pittsburgh, Pennsylvania, 15232, USA; 5 Department of Biostatistics, University of Pittsburgh, 310 Parran Hall, 130 DeSoto Street, Pittsburgh, Pennsylvania, 15261, USA; 6 Tobago Health Studies Office, Scarborough, Tobago, Trinidad and Tobago

**Keywords:** human herpesvirus 8, prostate cancer, case-cohort design

## Abstract

**Background:**

We previously reported a cross-sectional association between the presence of human herpesvirus 8 (HHV-8) serum antibodies and screen-detected prostate cancer in men living in Tobago. In the same study population, we examined the association between HHV-8 seropositivity and incident prostate cancer discovered at later screenings.

**Methods:**

In 40-81 year-old men without prostate cancer discovered at initial digital rectal examination (DRE) and prostate-specific antigen (PSA) screening, a case-cohort design measured the association between baseline HHV-8 seropositivity (modified immunofluorescence assay for antibodies against HHV-8 lytic antigens) and incident prostate cancer detected at DRE and PSA screenings three or five years later.

**Results:**

Analyses included 486 unique individuals, 96 incident prostate cancer cases, and 415 randomly selected subjects representing an at-risk cohort. By design, the random sub-cohort contained 25 incident prostate cancer cases. In the sub-cohort, the frequency of HHV-8 seropositivity increased across age groupings (40-49 years: 3.5%, 50-59 years: 13.6%, and ≥ 60 years: 22.9%). HHV-8 seropositivity was higher in men with elevated (≥ 4.0 ng/mL) than men with non-elevated PSA at initial screening (30.4% *vs*. 9.9% seropositive; crude odds ratio (OR) 3.96, 95% confidence interval (CI) 1.53-10.2; age-adjusted OR 2.42, 95% CI 0.91-6.47). HHV-8 seropositivity did not increase incident prostate cancer risk (age-adjusted hazard ratio (HR) 0.88, 95% CI 0.46-1.69).

**Conclusions:**

Case-cohort analysis did not identify association between HHV-8 seropositivity and incident prostate cancer. However, analyses uncovered possible association between HHV-8 and PSA (a marker of prostate inflammation). Co-occurrence of HHV-8 seropositivity and PSA elevation may explain cross-sectional association between HHV-8 and PSA screen-detected prostate cancer.

## Background

In 2008, prostate cancer was the fifth most common cancer and the sixth leading cause of cancer death among men worldwide [1]. Men of African descent experience higher prostate cancer incidence and mortality than any other racial group [1-5]. Other accepted risk factors include older age and family history. The otherwise poor understanding of prostate cancer etiology motivates search for specific causal agents. Though not consistently [6], studies find prostate cancer in association with infectious disease agents, including *Neisseria gonorrhoeae*, *Chlamydia trachomatis*, human papillomavirus (HPV) type 18, and *Treponema pallidum *(syphilis) [7-11]. Other studies find viral DNA or evidence of viral gene expression in prostate tissues (HPV, human herpes simplex virus type 2, cytomegalovirus, Epstein-Barr virus, and human herpesvirus 8 (HHV-8) [10,12-19]), stromal fibroblasts within prostate tumors (xenotropic murine leukemia virus-related virus or XMRV [20]), or malignant prostate epithelial cells (XMRV [21]). These infectious agents may elicit an immune response creating a cytokine tissue environment that leads to chronic inflammation, DNA damage, cellular proliferation, angiogenesis, and ultimately prostate cancer [10,13,22,23].

HHV-8, a DNA virus, causes Kaposi's sarcoma and primary effusion lymphoma. In a high prostate cancer risk cohort of African-Caribbean men living on Tobago [24,25], we found an association between HHV-8 seropositivity and prostate cancer discovered as a result of an initial prostate cancer screening (odds ratio [OR] 2.24, 95% confidence interval [CI] 1.29-3.90) [9]. Four studies completed later in other population settings could not confirm an association between HHV-8 and prostate cancer [6,11,26,27]. Therefore, our current study re-examines this association in our Tobago study population, through consideration of the association between HHV-8 seropositivity and prostate cancer discovered, not as a result of the initial screening, but later as a result of subsequent screenings.

## Methods

### Study Population

The Tobago Prostate Survey is an ongoing population-based longitudinal study of prostate cancer screening, as well as risk, in ≥ 40 year-old men living in Tobago [24]. Tobago is a small Caribbean island, 7 by 26 miles in size, with 8078 40-79 year-old men, according to a 2000 census [28]. The population as a whole is 89% African or Black and 7% mixed heritage by nationality or ethnicity [28]. Identification of study participants occurred through the agency of posters, flyers, public service announcements, public presentations, healthcare workers, private physicians, and word of mouth [24]. Prostate cancer screening occurred in three waves, Wave 1 - October 1997 to August 2003, Wave 2 - February 1999 to August 2003, and Wave 3 - May 2004 to March 2007. Although an open cohort, this report included only men screened at Wave 1 and subsequently rescreened at Waves 2 and/or 3. Study procedures included risk factor questionnaires, blood collections, and prostate cancer screening examinations, with prostate specific antigen (PSA) serum concentrations ≥ 4 ng/mL or abnormal digital rectal examinations (DRE) prompting referral for ultrasound-guided trans-rectal prostate biopsy [24].

**Figure 1:**
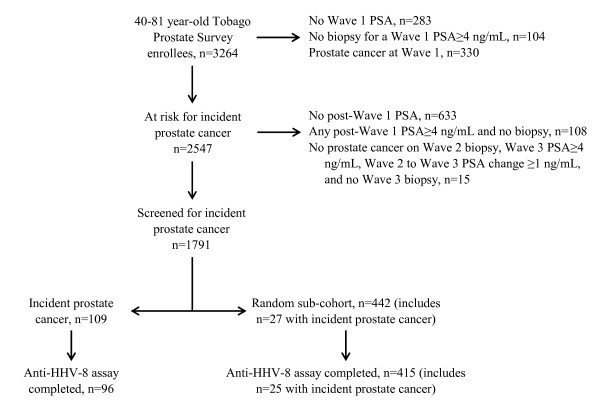
**Case-cohort design**. Study design features, identifying study exclusions, the study population screened for incident prostate cancer, the case group, and the random sub-cohort.

Wave 1 enrolled 3264 40-81 year-old men (97% self-reporting African descent). The current study excluded men missing Wave 1 PSA (n = 283), men with Wave 1 PSA ≥ 4.0 ng/mL not followed by prostate biopsy (n = 104), and men with prostate cancer detected at Wave 1 (n = 330), thereby leaving 2547 men at risk for prostate cancer at Wave 2 or Wave 3 (Figure 1). The study design excluded 756 at-risk men, including 633 at-risk men without subsequent PSA at either Wave 2 or Wave 3, 108 men with a Wave 2 or Wave 3 PSA ≥ 4.0 ng/mL not followed by biopsy, and 15 men with a prostate cancer negative Wave 2 biopsy, but no Wave 3 biopsy for Wave 3 PSA ≥ 4.0 ng/mL associated with ≥ 1.0 ng/mL PSA increase between Waves 2 and 3 (Figure 1). In the remaining 1791 at-risk men, a Wave 2 or Wave 3 biopsy completed before study closure (August 15, 2007) detected prostate cancer in 109 (Figure 1).

To measure the association between Wave 1 HHV-8 seropositivity and prostate cancer detection from a Wave 2 or Wave 3 screening, we used a case-cohort study design that compared prostate cancer cases at Wave 2 or Wave 3 (n = 109; Gleason 6 - 49%, Gleason 7 - 45%, Gleason 8 or 9 - 6%; pre-diagnostic screening PSA, mean 9.5 ng/mL, median 4.4 ng/mL) against a control group constructed as a simple random sample (n = 442) of the 1791 at-risk men screened for prostate cancer at Wave 2 or Wave 3. We used a case-cohort design because research costs prohibited determination of the HHV-8 status of all 1791 men in the at-risk group. As a result of the random selection procedure, 27 case men with prostate cancer at Wave 2 or Wave 3 entered the sub-cohort and contributed data as controls (Figure 1). The 442 and 1349 randomly selected and excluded men were statistically similar with respect to age, education, marital status, prostate cancer family history, history of smoking, personal history of cancer, history of benign prostatic hypertrophy, and Wave 1 PSA and DRE results. The 442 men selected for the sub-cohort survived a median 4.9 years (5^th^-95^th ^percentile 1.7-6.8 years) between Wave 1 and the last complete post-Wave 1 visit. For the 109 cases, a median 7.2 months (25^th^-75^th ^percentile 2.5-13.8 months) elapsed between pre-diagnostic screening and confirmatory biopsy.

Study participants signed an informed consent approved by the Institutional Review Boards of the Tobago Division of Health and Social Services and the University of Pittsburgh.

### Laboratory Methods

Laboratory assays used frozen serum samples (thawed once and never re-frozen) stored temporarily in a -20°C freezer at the Tobago Health Studies office in Scarborough, Tobago, and stored later in a -80°C freezer at the University of Pittsburgh, Department of Epidemiology. PSA measurements used either Abbott Diagnostics AxSYM^® ^or Siemens Healthcare Diagnostics ADVIA Centaur^® ^immunoassays. To detect serum antibodies against HHV-8 lytic antigens, an indirect immunofluorescence assay, as described elsewhere [29], used BCBL-1 cells containing the HHV-8 genome with a modified Rta gene inducible by doxycyclin [30]. Targeting fixed and permeabilized B cells that have been induced to replicate HHV-8, this assay potentially identifies any of the lytic proteins involved in HHV-8 replication. A single reader (FJJ), blinded to samples' prostate cancer case status, examined microscopic slides for fluorescence. A positive assay result required specific fluorescence at a 1:100 dilution. Each assay run included prostate cancer case and non-case subject sera and known HHV-8 positive and negative control sera. We tested each sample in duplicate on separate days with disagreements resolved by retesting on a third day. Estimates of assay sensitivity and specificity range between 53.4-89.9% and 96.6-97.5%, respectively [31]. Kappa agreement between first and second assay results was 0.76 (95% CI 0.71-0.81).

### Data Analysis

We used the chi-square test to evaluate 1) the statistical significance of differences between at-risk men with incomplete *vs*. complete follow-up with respect to baseline factors such as age, education, marital status, family history of prostate cancer, history of smoking, cancer, and benign prostatic hypertrophy, and results from PSA and DRE screening and 2) the statistical significance of differences in the sub-cohort rates of HHV-8 seropositivity with respect to the same baseline factors. In analyses restricted to sub-cohort members, logistic regression estimated odds ratios to express strengths of association between Wave 1 PSA elevation and Wave 1 HHV-8 seropositivity, two factors determined at the same point in time. We used Cox proportional hazards models (Breslow weighted denominator method) for case-cohort designs to estimate hazard ratio [HR] measures of unadjusted and age-adjusted association between HHV-8 seropositivity measured at baseline (Wave 1) and prostate cancer detected later in time, at Wave 2 or Wave 3 [32]. These models compare prostate cancer cases detected at one or the other points in time (Wave 2 or Wave 3) with the appropriate risk set, constructed from sub-cohort members disease-free and available at Wave 2 or Wave 3. For prostate cancer cases in the sub-cohort, Cox models started follow-up at the Wave 1 screening date and censored follow-up at the Wave 2 or Wave 3 screening date that led to prostate cancer diagnosis. For non-cases in the sub-cohort, Cox models started follow-up at the Wave 1 screening date and censored follow-up at the date of the last completed Wave 2 or Wave 3 screening. Calculating age on the date of the Wave 1 PSA blood collection, age adjustments used either two age categories (40-59 and ≥ 60 years), three age categories (40-49, 50-59, or ≥ 60 years), four age categories (40-44, 45-49, 50-59, and ≥ 60 years), or age (integer years) modeled as a continuous variable. Statistical inferences used a p = 0.05 two-sided significance level.

## Results

Table 1 summarizes the characteristics of all 3264 40-81 year-old men enrolled at Wave 1. The current study excluded 717 men missing a Wave 1 PSA, men with Wave 1 PSA ≥ 4.0 ng/mL not followed by prostate biopsy, and men with prostate cancer detected at Wave 1, leaving 2547 screen-negative men at risk for prostate cancer at Wave 2 or Wave 3 (Figure 1). Table 1 compares these 2547 at-risk men with the 717 men who were either inadequately screened at Wave 1 or discovered to have prostate cancer at Wave 1. At-risk men were younger and better educated (Table 1). At-risk men less often reported a history of smoking, cancer, or benign prostatic hypertrophy (Table 1). PSA values were lower and DRE results positive less often in at-risk men, as expected, since the group not at risk included men with prostate cancer detected as direct result of PSA elevation or DRE abnormality (Table 1). The study design excluded 756 men from the group of 2547 at-risk men eligible for follow-up (Figure 1). As shown in Table 1 the 756 at-risk men with incomplete follow-up differed from the 1791 men with complete follow-up, as follows, age (35.6% *vs*. 26.0% ≥ 60 years), smoking history (48.5% *vs*. 39.6%), personal cancer history (1.3% *vs*. 0.3%), Wave 1 PSA ≥ 4 ng/mL (13.5% *vs*. 5.5%), and Wave 1 DRE (64.7% *vs*. 76.3% negative and 20.0% *vs*. 10.3% missing).

**Table 1:** Characteristics of all men enrolled at Wave 1, men not at risk and at risk for prostate cancer at Wave 2 or Wave 3 based on Wave 1 screen results, and at-risk men with incomplete and complete follow-up.

			Risk status after Wave 1				Follow-up				
				
	All		Not at risk		At risk		Incomplete		Complete		
	n = 3264		n = 717		n = 2547		n = 756		n = 1791		
				
Characteristic	n^1^	%	n^1^	%	n^1^	%	n^1^	%	n^1^	%	p-value^2^
*Demographic*											
Age (years)											<.0001
40-44	624	19.1	59	8.2	565	22.2	151	20.0	414	23.1	
45-49	571	17.5	71	9.9	500	19.6	115	15.2	385	21.5	
50-59	914	28.0	166	23.2	748	29.4	221	29.2	527	29.4	
60-81	1155	35.4	421	58.7	734	28.8	269	35.6	465	26.0	
Education	(23)		(7)		(16)		(7)		(9)		0.76
≤11 years	2411	74.4	553	77.9	1858	73.4	553	73.8	1305	73.2	
12+ years	830	25.6	157	22.1	673	26.6	196	26.2	477	26.8	
Marital status	(26)		(10)		(16)		(7)		(9)		0.13
ever married	2669	82.4	590	83.5	2079	82.1	602	80.4	1477	82.9	
never married	569	17.6	117	16.5	452	17.9	147	19.6	305	17.1	
*Family history of ..*.											
Prostate cancer											0.02
missing	323	9.9	77	10.7	246	9.7	86	11.4	160	8.9	
yes	218	6.7	40	5.6	178	7.0	64	8.5	114	6.4	
no	2723	83.4	600	83.7	2123	83.4	606	80.2	1517	84.7	
*History of ..*.											
Smoking	(21)		(7)		(14)		(3)		(11)		<.0001
yes	1410	43.5	341	48.0	1069	42.2	365	48.5	704	39.6	
no	1833	56.5	369	52.0	1464	57.8	388	51.5	1076	60.4	
Cancer	(43)		(13)		(30)		(9)		(21)		0.002
yes	26	0.8	11	1.6	15	0.6	10	1.3	5	0.3	
no	3195	99.2	693	98.4	2502	99.4	737	98.7	1765	99.7	
Benign prostatic hypertrophy	(84)		(24)		(60)		(17)		(43)		0.67
yes	248	7.8	78	11.3	170	6.8	53	7.2	117	6.7	
no	2932	92.2	615	88.7	2317	93.2	686	92.8	1631	93.3	
*Entry prostate cancer screen*											
PSA (ng/mL)	(190)		(190)								<.0001
0.0-0.9	1213	39.5	43	8.2	1170	45.9	306	40.5	864	48.2	
1.0-1.9	850	27.7	45	8.5	805	31.6	206	27.2	599	33.4	
2.0-2.9	268	8.7	21	4.0	247	9.7	84	11.1	163	9.1	
3.0-3.9	150	4.9	26	4.9	124	4.9	58	7.7	66	3.7	
4.0-9.9	354	11.5	199	37.8	155	6.1	73	9.7	82	4.6	
10+	239	7.8	193	36.6	46	1.8	29	3.8	17	0.9	
DRE											<.0001
missing	555	17.0	220	30.7	335	13.2	151	20.0	184	10.3	
positive	636	19.5	279	38.9	357	14.0	116	15.3	241	13.5	
negative	2073	63.5	218	30.4	1855	72.8	489	64.7	1366	76.3	

PSA - prostate-specific antigen, DRE - digital rectal examination

1. Numbers in parentheses indicate missing data

2. Statistical significance (chi-square) of differences between at-risk men with incomplete and complete follow-up

Wave 1 HHV-8 serologic status was available for 415 (93.9% of 442) men in the sub-cohort and for 96 (88.1% of 109) men in the case group (Gleason 6 - 49%, Gleason 7 - 45%, Gleason 8 or 9 - 6%; pre-diagnostic PSA, mean 10.0 ng/mL and median 4.4 ng/mL). Table 2 summarizes the baseline characteristics for these men with non-missing HHV-8. In addition, Table 2 compares sub-cohort rates of HHV-8 seropositivity according to the same baseline characteristics. Referenced against the sub-cohort, characteristics of the case group included older age, less frequent smoking history, more frequent benign prostatic hypertrophy history, more frequently elevated Wave 1 PSA (27.1% *vs*. 5.5% PSA ≥ 4 ng/mL), and more frequently positive Wave 1 prostate cancer screening (43.8% *vs*. 16.9% DRE or PSA positive). Case and sub-cohort HHV-8 seropositivity rates were 17.7% and 11.1%, respectively. When compared with 40-49 year-old sub-cohort men (3.5% HHV-8 seropositive), HHV-8 seropositivity was higher in 50-59 year-old sub-cohort men (13.6% HHV-8 seropositive) and higher yet in ≥ 60 year-old sub-cohort men (22.9% HHV-8 seropositive). HHV-8 seropositivity rates were lower in sub-cohort men with a history of smoking than those without (7.1% *vs*. 13.9%) and higher in sub-cohort men with a history of benign prostatic hypertrophy than those without (24.0% *vs*. 10.5%). HHV-8 seropositivity increased with Wave 1 PSA (7.6%, 12.1%, 12.7%, and 30.4% for PSA 0.0-0.9, 1.0-1.9, 2.0-3.9, and ≥ 4 ng/mL, respectively). HHV-8 seropositivity was higher in men with elevated (≥ 4.0 ng/mL) than men with non-elevated PSA (30.4% *vs*. 9.9% seropositive; crude OR 3.96, 95% CI 1.53-10.2; age-adjusted OR 2.42, 95% CI 0.91-6.47; data not shown). HHV-8 seropositivity was higher in sub-cohort men with a positive than in men with a negative Wave 1 prostate cancer screen result (20.0% *vs*. 9.3% seropositive).

**Table 2:** Case and sub-cohort group characteristics, with sub-cohort rate of HHV-8 seropositivity, according to baseline characteristic

	Cases (n = 96)		Sub-cohort (n = 415)		Sub-cohort HHV-8 Pos		
		
Characteristic	n^1^	Col %	n^1^	Col %	n^1^	Row %	p-value^2^
*Demographic*							< .0001
Age (years)							
40-49	12	12.5	200	48.2	7	3.5	
50-59	37	38.5	110	26.5	15	13.6	
60-81	47	49.0	105	25.3	24	22.9	
Education			(2)		(0)		0.83
≤11 years	77	80.2	318	77.0	36	11.3	
12+ years	19	19.8	95	23.0	10	10.5	
Marital status							0.48
ever married	82	85.4	336	81.0	39	11.6	
never married	14	14.6	79	19.0	7	8.9	
*Family history of ..*.							
Prostate cancer							0.80
missing	5	5.2	32	7.7	4	12.5	
yes	5	5.2	27	6.5	2	7.4	
no	86	89.6	356	85.8	40	11.2	
*History of ..*.							
Smoking			(1)		(0)		0.029
yes	34	35.4	170	41.1	12	7.1	
no	62	64.6	244	58.9	34	13.9	
Benign prostatic hypertrophy	(3)		(8)		(0)		0.039
yes	10	10.8	25	6.1	6	24.0	
no	83	89.2	382	93.9	40	10.5	
*Entry prostate cancer screen*							
PSA (ng/mL)							0.010
0.0-0.9	7	7.3	197	47.5	15	7.6	
1.0-1.9	27	28.1	140	33.7	17	12.1	
2.0-3.9	36	37.5	55	13.3	7	12.7	
≥ 4.0	26	27.1	23	5.5	7	30.4	
DRE							0.34
missing	4	4.2	48	11.6	4	8.3	
positive	29	30.2	54	13.0	9	16.7	
negative	63	65.6	313	75.4	33	10.5	
DRE positive and/or PSA ≥ 4 ng/mL							0.009
yes	42	43.8	70	16.9	14	20.0	
no	54	56.3	345	83.1	32	9.3	
*Study-specific*							
HHV-8 sero-status							
positive	17	17.7	46	11.1			
negative	79	82.3	369	88.9			

PSA - prostate-specific antigen, DRE - digital rectal examination, HHV-8 - human herpesvirus 8

1. Numbers in parentheses indicate missing data

2. Statistical significance (chi-square) of differences in HHV-8 seropositivity according to baseline characteristic

Table 3 compares Wave 1 HHV-8 seropositivity between the case and sub-cohort groups, according to age and Wave 1 PSA. Age-specific HHV-8 seropositivity rates were lower in case than sub-cohort men (40-49 years: 0.0% *vs*. 3.5% and 50-59 years: 10.8% *vs*. 13.6%), except in the oldest age group (≥ 60 years: 27.7% *vs*. 22.9%). In men with a non-elevated (< 4 ng/mL) Wave 1 PSA, the HHV-8 seropositivity rate was higher in the case group (17.1% *vs*. 9.9%). In men with an elevated (≥ 4 ng/mL) Wave 1 PSA, however, the HHV-8 seropositivity rate was lower in the case group (19.2% *vs*. 30.4%). In the two age sub-groups with appreciable HHV-8 seropositivity, age-specific HHV-8 seropositivity rates were not consistently higher or lower in case than sub-cohort men with non-elevated Wave 1 PSA (50-59 years: 11.5% *vs*. 13.7% and ≥ 60 years: 28.1% *vs*. 20.4%) and consistently lower in case than sub-cohort men with elevated Wave 1 PSA (50-59 years: 9.1% *vs*. 12.5% and ≥ 60 years: 26.7% *vs*. 41.7%).

**Table 3:** Sub-cohort and case group Wave 1 HHV-8 seropositivity, by age and Wave 1 PSA result

		Cases			Sub-cohort		
		
Age years	PSA ng/mL		HHV-8 positive			HHV-8 positive	
					
		n	n	%	n	n	%
40-49	All	12	0	0.0	200	7	3.5
50-59	All	37	4	10.8	110	15	13.6
≥ 60	All	47	13	27.7	105	24	22.9
							
All	< 4	70	12	17.1	392	39	9.9
All	≥ 4	26	5	19.2	23	7	30.4
							
40-49	< 4	12	0	0.0	197	6	3.0
50-59	< 4	26	3	11.5	102	14	13.7
≥ 60	< 4	32	9	28.1	93	19	20.4
							
40-49	≥ 4				3	1	33.3
50-59	≥ 4	11	1	9.1	8	1	12.5
≥ 60	≥ 4	15	4	26.7	12	5	41.7

PSA - prostate-specific antigen, DRE - digital rectal examination, HHV-8 - human herpesvirus 8

Table 4 shows associations, unadjusted and age-adjusted, between Wave 1 HHV-8 seropositivity and prostate cancer at Wave 2 or Wave 3, overall and in sub-groups defined by Wave 1 screen results. Though not statistically significant, HR point estimates indicate lower prostate cancer risk in HHV-8 seropositive men, overall (age-adjusted HR 0.88, 95% CI 0.46-1.69) and in HHV-8 seropositive men with elevated Wave 1 PSA (age-adjusted HR 0.39, 95% CI 0.10-1.63), and equivalent risk in HHV-8 seropositive men with non-elevated Wave 1 PSA (age-adjusted HR 1.03, 95% CI 0.49-2.16). In men eligible for prostate biopsy at Wave 1 (DRE or PSA positive), HHV-8 seropositivity reduced risk (age-adjusted HR 0.59, 95% CI 0.18-1.91) to a statistically insignificant level. In perhaps the most meaningful sub-group, men not eligible for prostate biopsy at Wave 1 (DRE not positive and PSA < 4.0 ng/mL), analyses supplied no evidence of association between seropositivity and prostate cancer risk (age-adjusted HR: 1.02, 95% CI 0.44-2.39). In men with non-elevated Wave 1 PSA, single-year-of-age-adjusted (continuous) risk estimates were HR 0.80 (95% CI 0.19-3.34) and HR 1.27 (95% CI 0.50-3.25) for the 50-59 and ≥ 60 year-old men, respectively (data not shown). Using all (96 case and 415 sub-cohort) men or only ≥ 45 year-old (95 case and 312 sub-cohort) men made no meaningful difference in the age-adjusted risk estimates (data not shown).

**Table 4:** Unadjusted and age-adjusted associations (hazard ratio) between Wave 1 HHV-8 seropositivity and prostate cancer at Wave 2 or Wave 3, overall and within strata defined by Wave 1 prostate cancer screening test results

Wave 1 prostate cancer screening test result	Cases		Sub-cohort		Unadjusted		Age-adjusted	
	
	Pos	n	Pos	n	HR	95% CI	HR	95% CI
Overall	17	96	46	415	1.46	0.78-2.74	0.88^1^	0.46-1.69
PSA (ng/mL)								
< 4	12	70	39	392	1.61	0.77-3.38	1.03^1^	0.49-2.16
≥ 4	5	26	7	23	0.44	0.11-1.71	0.39^2^	0.10-1.63
DRE positive or PSA ≥ 4 ng/mL								
no	9	54	32	345	1.67	0.72-3.86	1.02^1^	0.44-2.39
yes	8	42	14	70	0.85	0.52-2.38	0.59^3^	0.18-1.91

Pos - number HHV-8 positive, n - case or sub-cohort count, HR - hazard ratio, CI - confidence interval, PSA - prostate-specific antigen, DRE - digital rectal examination

1. Adjusted across four age categories: 40-44, 45-49, 50-59, ≥ 60 years

2. Adjusted across two age categories: 40-59, ≥ 60 years

3. Adjusted across three age categories: 40-49, 50-59, ≥ 60 years

## Discussion

Our previous study used an immunofluorescence assay to measure HHV-8 antibodies in 138 prostate cancer cases and in 140 age-matched controls [9]. HHV-8 seropositivity was significantly more frequent in cases than controls (39.9% vs. 22.9%, OR 2.24, 95% CI 1.29-3.90) [9]. Our previous study compared Wave 1 screen-detected (DRE positive and/or PSA elevated) prostate cancer cases with DRE negative and PSA non-elevated controls. In the same Tobago study population, using a similar assay, the current prospective case-cohort study offered an opportunity to evaluate temporal relationships between HHV-8 seropositivity and prostate cancer, in men with and without elevated PSA at baseline. Including men with non-elevated (< 4.0 ng/mL) Wave 1 PSA and men with elevated (≥ 4.0 ng/mL) Wave 1 PSA, but prostate cancer not seen on Wave 1 biopsy, case-cohort analysis did not observe HHV-8-related incident prostate cancer risk in men overall (age-adjusted HR 0.88, 95% CI 0.46-1.69), in men with Wave 1 PSA < 4 ng/mL (age-adjusted HR 1.03, 95% CI 0.49-2.16), or in men without a positive Wave 1 prostate cancer screen result (age-adjusted HR 1.02, 95% CI 0.44-2.39; Table 4).

A positive association between HHV-8 seropositivity and prevalent prostate cancer in a cross-sectional study [9] and an inverse (though not statistically significant) association in a prospective study, an inverse association most evident in men sent for biopsy (*e.g*., PSA ≥ 4 ng/mL, age-adjusted HR 0.39, 95% CI 0.10-1.63; Table 4), lead to the following speculation. HHV-8 may associate with factors, such as elevated PSA, that prompt biopsy and subsequent recognition of prostate cancer. In effect, HHV-8 may segregate men with manifest and emergent prostate cancer into two groups, HHV-8 seropositive prostate cancer detected immediately and HHV-8 seronegative prostate cancer detected later. This selection bias may explain opposing positive and negative HHV-8 associations seen with prevalent and incident prostate cancer, respectively. A similar selection bias may explain inverse associations between HHV-8 and prostate cancer observed in other prospective studies, as described below.

Four comparative studies of HHV-8 and prostate cancer have appeared [6,11,26,27] since our 2004 publication [9]. In a prospective study from Finland, ELISA detected serum antibodies against the HHV-8 ORF65 protein in 3 (1.8%) of 163 men with incident prostate cancer and in 7 (2.4%) of 288 age-matched men without cancer (OR 0.74, 95% CI 0.19-2.88; [27]). In a U.S. population-based case-control study, the immunofluorescence assay detected serum antibodies against HHV-8 lytic antigens less often in cases than controls (95 African-American cases and 75 controls: OR 0.56, 95% CI 0.28-1.14; 104 white cases and 80 controls: OR 0.71, 95% CI 0.36-1.43; [26]). In a study of 691 individually matched case-control pairs nested within the U.S. Health Professional Follow-up Study, the immunofluorescence assay detected plasma antibodies against lytic antigens less often in men diagnosed with prostate cancer, on average, 3.1 years later (OR 0.70, 95% CI 0.52-0.95; [6]). Finally, in the Prostate, Lung, Colorectal, and Ovarian (PLCO) Cancer Screening Trial, ELISA detected IgG antibodies against the HHV-8 K8.1 structural protein in study entry serum samples from 103 (13.5%) of 765 and 103 (11.3%) of 915 white prostate cancer cases and age-matched controls, respectively (OR 1.3, 95% CI 0.9-1.7) and in 2 (1.9%) of 103 and 22 (6.0%) of 367 black cases and age-matched controls, respectively (OR 0.3, 95% CI 0.1-1.4; [11]). On balance, these studies suggest that HHV-8 does not influence prostate cancer risk.

Analyses restricted to the sub-cohort showed strong association 1) between HHV-8 seropositivity and increasing age, a result also seen in Tobago women [33] and many other populations [34,35], and 2) between HHV-8 seropositivity and PSA elevation ≥ 4.0 ng/mL. Though not statistically significant (p = 0.17), age-adjusted geometric mean Wave 1 PSA was 18% higher in HHV-8 seropositive than seronegative sub-cohort men (data not shown). The age-adjusted odds of HHV-8 seropositivity was more than two-fold higher in sub-cohort men with elevated PSA than men with non-elevated PSA. Personal or environmental factors related to HHV-8 exposure or immune function may explain the age association with HHV-8 [33]. Accepting PSA as a marker of prostate inflammation, we postulate that the association between HHV-8 seropositivity and elevated PSA signifies either the effects of HHV-8 infection on prostate inflammation [19] or the effects of prostate inflammation on HHV-8 reactivation. PSA elevation has been observed in relation to other infectious disease agents [36,37].

Study strengths include unique population and setting (predominantly African ancestry Tobago residents [38]) and a control group large enough to estimate age-specific HHV-8 seroprevalence rates with acceptable precision. Study limitations include unavoidable misclassification according to prostate cancer outcome. DRE and PSA invariably miss instances of biopsy detectable prostate cancer. The Prostate Cancer Prevention Trial, for example, observed a 15% prostate cancer biopsy prevalence in men with PSA ≤4 ng/mL [39]. In addition, our study can not define the prostate cancer risk experience of men who did not return for repeat screening. Follow-up intervals much longer than our five-year interval between initial and final screening may be needed to detect a prostate cancer effect from any chronic inflammation caused by HHV-8 infection. Also, HHV-8 may cause inflammation and prostate cancer only in a relatively small genetically susceptible sub-group. Finally, a small case count limits, especially in sub-groups, the precision of our risk estimates. For example, in men not eligible for prostate biopsy at Wave 1, the 95% confidence interval embraced both 50% lower and 200% higher prostate cancer risks in relation to HHV-8 seropositivity.

## Conclusions

Our prospective study could not demonstrate an association between HHV-8 seropositivity and incident prostate cancer. However, analyses uncovered a strong relationship between elevated HHV-8 seropositivity and PSA. The HHV-8 association previously observed with prevalent prostate cancer may signify enhanced detection of prostate cancer possibly caused by the effects of HHV-8 on PSA. In this context, the association we observed between HHV-8 seropositivity and PSA elevation deserves further study.

## List of Abbreviations

HHV-8: human herpesvirus 8; DRE: digital rectal examination; PSA: prostate-specific antigen; OR: odds ratio; CI: confidence interval; HR: hazards ratio; HPV: human papillomavirus; PLCO: Prostate Lung Colorectal, and Ovarian Cancer Screening Trial.

## Competing interests

The authors declare that they have no competing interests.

## Authors' contributions

ACM participated in study design, data analysis and interpretation, prepared a first draft of the manuscript, and helped revise the final manuscript. FJJ completed immunofluorescence assays and helped revise the final manuscript. CHB conceived the study, acquired data, provided study coordination, and helped revise the final manuscript. JWW selected analytic methods and directed statistical analysis. ALP acquired data and provided study coordination. JLW participated in study design, data analysis and interpretation and helped revise the final manuscript. All authors read and approved the final manuscript.
